# Tailor-designed nanoparticle-based PdNiSn catalyst as a potential anode for glycerol fuel cells

**DOI:** 10.1038/s41598-023-40374-4

**Published:** 2023-08-15

**Authors:** Ghada H. El-Nowihy

**Affiliations:** Chemical Engineering Department, Faculty of Engineering, The British University in Egypt, Cairo, 11837 Egypt

**Keywords:** Catalysis, Electrochemistry, Energy, Green chemistry

## Abstract

In order to effectively use glycerol as a fuel in direct glycerol fuel cells, a catalyst that can break the C–C bond and enhance the electro-oxidation of glycerol to CO_2_ is necessary. In this particular investigation, a palladium-nickel-tin nanocomposite electrodeposited on a glassy carbon electrode (PdNiSn/GC) exhibited excellent activity towards the electro-oxidation of glycerol, thanks to the synergistic effect of the catalyst composition. The PdNiSn/GC surface generated a peak current (*I*_p_) that was 2.5 times higher than that obtained at a Pd/GC electrode, with a cathodic shift in the onset potential (*E*_onset_) of approximately 300 mV. Additionally, the current obtained at the PdNiSn/GC surface remained stable during continuous electrolysis. Capacitance measurements were used to interpret the results of the electrocatalytic activity, and high-performance liquid chromatography indicated that the products of the glycerol electro-oxidation reaction were oxalic acid and formic acid, which were subsequently oxidized to CO_2_, as revealed by the charge calculations. The results depict that the synergy between Pd, β-Ni(OH)_2_, and SnO_2_ is crucial for boosting GEOR through enhancing the C–C bond cleavage and completely oxidize the reaction intermediates to CO_2_.

## Introduction

The continuous expansion in commercial activities and the gradual rising in population growth have directly increased the energy demand and necessitated finding alternative energy resources to fossil fuels. Amongst, the efficient utilization of chemical fuels in fuel cells has a great potential to contribute in an efficient and CO_2_-zero emission energy systems^1^.

Considering the different types of fuel cells, the polymer electrolyte membrane fuel cells offer numerous benefits over the other types of fuel cells, e.g.; high energy density, quick energy release; insensitivity to CO_2_ allowing the use of air as an oxidant, and operating at moderate temperatures (75–150 °C)^2,3^.

The use of alcohols, in particular glycerol, in the direct alcohol fuel cell is an interesting target for the fuel in such a scheme. Glycerol electrooxidation reaction (GEOR) could take place at the surface of electrocatalysts through the oxidation of its hydroxyl groups in order to generate electrical energy^4^. Furthermore, GEOR produces high theoretical energy density of 6 kWh kg^−1^, it is non-flammable and non-volatile^5^, and has low crossover rate through the membrane when compared to methanol^6^. In addition, the selective GEOR could produce value-added intermediates. Thus, another promising aspect of GEOR is the electrosynthesis of value-added molecules such as; glyceric acid, tartronic acid, mesoxalate, and 1,3-dihydroxyacetone^7–9^.

Glycerol is a common product from biodiesel industry which produces approximately hundreds of millions of kilograms of glycerol each year making it an available and low-price fuel^10–13^. In addition, glycerol is considered a good source for hydrogen production^14,15^. Thermodynamics depict that glycerol electro-oxidation reaction (GEOR) in the anodic compartment of DGFCs is energetically more efficient than oxygen evolution reaction (OER) in the electrolysis cell for the production of hydrogen^9,10^. GEOR takes place in the anodic compartment of the DGFCs producing protons and carbon dioxide while protons are reduced in the cathodic compartment producing hydrogen as shown in Eqns. 1–3^11^.

1 $$ C_{3} H_{8} O_{3} + 3H_{2} O \rightleftarrows 3CO_{2} + 14H^{ + } + 14e^{ - } \quad {\text{Anodic}}\;{\text{reaction}} $$

2 $$ 14H^{ + } + 14e^{ - } \rightleftarrows 7H_{2} \quad {\text{Cathodic}}\;{\text{reaction}} $$

3 $$ C_{3} H_{8} O_{3} + 3H_{2} O \rightleftarrows 3CO_{2} + 7H_{2} \;\left( {E_{cell}^{o} = 0.003 V} \right)\quad {\text{Overall}}\;{\text{reaction}} $$

However, the efficient and complete oxidation of glycerol into CO_2_ in the direct glycerol fuel cells (DGFCs) is still a great challenge as this process is multielectron and multiproton process which requires C–C bond cleavage (major challenge in electrocatalysis).

Pt electrocatalyst was considered to be the most efficient catalyst for the DGFC due to its high activity^16–19^, however, the production of the poisoning CO intermediate at its surface during the oxidation scheme of glycerol along with its high cost has limited its use^16,20,21^. This has motivated the use of alternative electrocatalysts, e.g., Pd, Ag, and Ni in order to boost the performance of the DGFC. In this regard, Pd-based electrocatalysts are considered a good candidate for the DGFC due to their advantages over the Pt-based electrocatalysts^16^. Pd shows better activity towards alcohols oxidation in the alkaline medium than Pt does^22–25^. In addition, the chance of the electrode poisoning by adsorbed carbon monoxide (CO_ads_) in alkaline medium is less than the acidic medium as the chemisorbed intermediates weakly bonds to the catalyst surface and the amount of poisoning species is less in alkaline medium^26^.

A lot of work was done to improve the effectiveness of Pd catalyst towards glycerol electro-oxidation, this includes adding another metal to Pd surface in order to increase its activity. It also includes, the employment of high surface area carbon materials as electrode material, e.g., carbon nanotubes (CNTs), activated carbon, and carbon nanofibers due to their good physiochemical properties and good resistance to corrosion^27^.

The current study focuses on tailoring and designing an efficient electro-catalyst for GEOR. Employing noble metal-based materials for GEOR could subject them to be compromised by the strongly adsorbed CO poison^28,29^. Thus, adding other catalysts is intended to increase the catalyst’s tolerance towards CO poisoning based on bifunctional and electronic effects^30^.

In this study, PdNiSn nanocomposite is electrodeposited at the glassy carbon (GC) electrode surface utilizing a simple electrochemical method then examined towards GEOR. The synergy between the component of the catalyst is believed to introduce a uniform dispersion of the nanoparticles and thus increase the number of active sites available for OH^−^ adsorption/desorption, GEOR, and the oxidation of CO. This work will highlight the role of Pd in offering a suitable surface for the glycerol molecules adsorption and oxidation, and the role of Sn in making the Ni quickly restore its highly active state.

The selection of the catalyst and the preparation method are believed to introduce a structural organization that facilitates the oxidation of the glycerol and the reaction intermediates to CO_2_ and suppress the formation of the poisoning CO. It is aimed to evaluate the effect of incorporating Ni and Sn atoms/oxides in the catalyst structure on the performance of GEOR. Not only that, the catalyst surface will be proved to be suitable for energy storage applications through evaluating its real capacitance.

## Experimental

### Electrodes, pre-treatments, and measurements

In this study, a three-electrode electrochemical cell was utilized. The working electrode was a glassy carbon electrode (diameter = 3.0 mm), while the reference electrode was Ag/AgCl/KCl (saturated), and a spiral Pt wire was used as the counter electrode. Prior to use, the GC electrode was smoothed using alumina (0.5 μm down to 0.05 μm), then rinsed successively with distilled water and sonicated in ethanol and water^31–34^. It is important to note that all potentials were converted from Ag/AgCl to RHE using the following equation:

4 $$  E_{{{\text{RHE}}}}  = E_{{\left( {{\text{Ag}}/{\text{AgCl}}} \right)}}  + 0.059\;{\text{pH}} + E_{{0\left( {{\text{Ag}}/{\text{AgCl}}} \right)}}   $$

where *E*_*o*(Ag/AgCl)_ = 0.1976 V at 25° C and *E*_(Ag/AgCl)_ is the working potential.

The chemicals used were supplied from Sigma-Aldrich and the solutions were prepared using double distilled water.

A VersaSTAT 4 potentiostat operated with VersaStudio software was used for the electrochemical measurements which were all performed at room temperature. The electrochemical measurements of glycerol electro-oxidation reaction (GEOR) were performed in 0.1 M KOH containing 0.3 M glycerol (no iR-correction).

Electrochemical impedance spectroscopy (EIS) measurements were conducted at open circuit potential within the frequency range from 100 to 30 mHz. Then, EIS curves (*Z*_re_ − *Z*_im_) were turned into capacitance curves (*C*_re_ − *C*_im_) using VersaStudio software. The equivalent circuit diagram for this system is shown in Fig. 4, with the inset indicating the charge transfer resistance (*R*_ct_) of the electrode/electrolyte interface associated with GEOR, the solution resistance (*R*_s_), and constant phase element (*CPE*) of the double layer capacitance, respectively.

### Electrode’s modification

To deposit the PdNiSn nanocomposite on the surface of the GC electrode, potential step electrolysis was used, with the potential ranging from 0 to − 1 V vs. Ag/AgCl/KCl (saturated) for a duration of 3 min. The electrolysis was performed using a solution of 50 mM of NaNO_3_ (purged with N_2_), which contained 1 mM of each metal ion salt^35^. It should be noted that SnCl_2_ can slowly hydrolyze to form Sn(OH)Cl, resulting in a slightly milky appearance of the solution, indicating the formation of Sn(OH)Cl.

### Materials characterization

The characterization of the morphology and composition of the prepared catalysts were performed using a field-emission scanning electron microscope (FE-SEM, FEGESEM, model Quattro S, supplied by Thermo Scientific USA) joined with energy dispersive X-ray spectroscopy (EDS, EDAX genitive). The structures of Pd and PdNiSn nanocomposite crystals were characterized using X-ray diffraction (XRD, PANalytical, X’Pert PRO) operated with Cu target, l = 1.54 Å, and scan speed = 0.05°/s. The products formed as a result of GEOR at PdNiSn/GC electrode were identified utilizing a high performance liquid chromatography (HPLC, Aglient 1100) integrated with a diode array detector at wavelength of 228 nm. The incorporated column was μ Bondapak C18 10 μm 125 A with an eluent 0.1% phosphoric acid (flow rate = 0.5 mL min^−1^).

## Results and discussion

### Materials characterization

**Figure 1:**
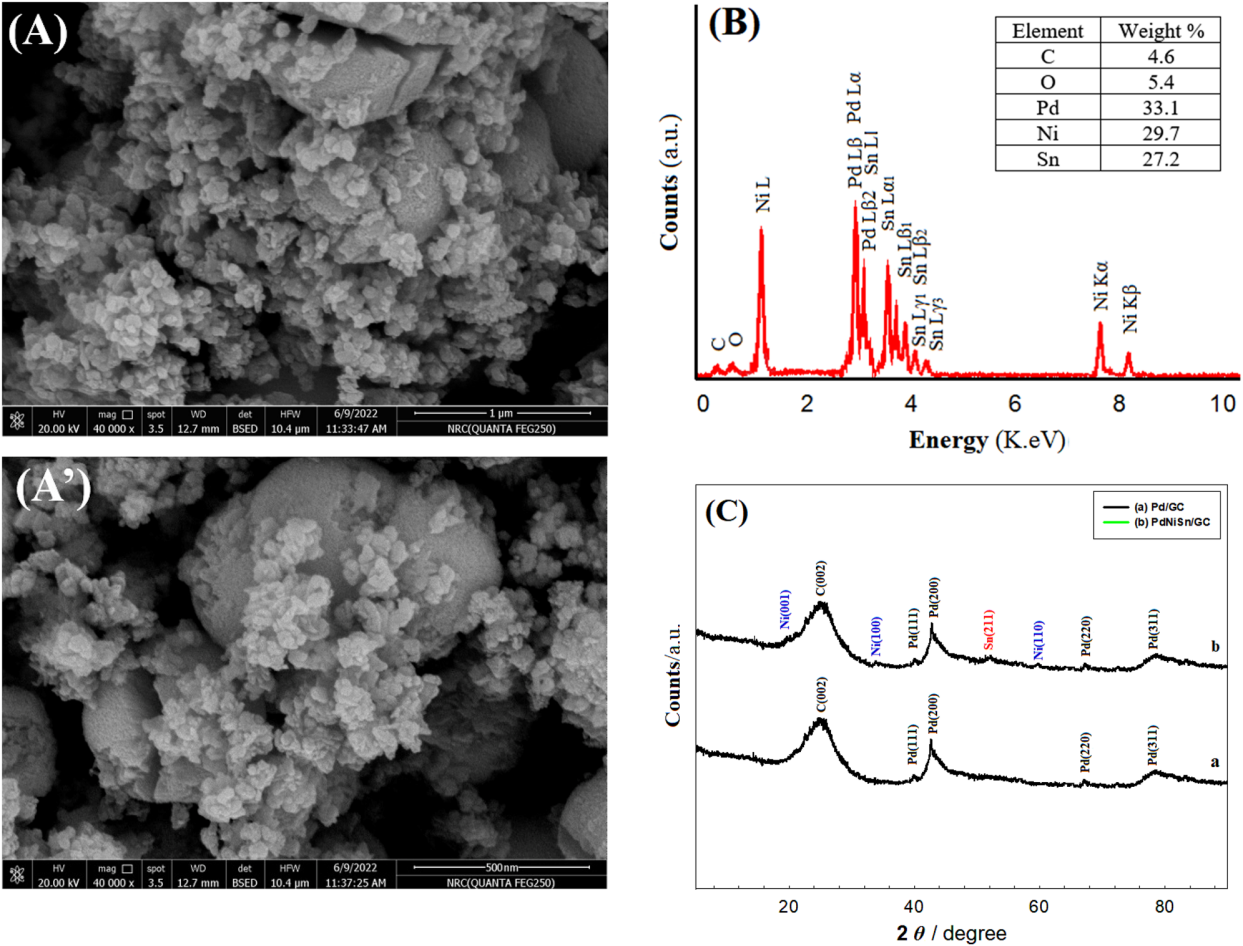
(**A**, **A’**) FE-SEM images before and after the reaction, (**B**) EDS spectrum, and (**C**) XRD pattern of PdNiSn/GC electrode.

Figure 1A and 1A’ show the FE-SEM images of PdNiSn/GC electrode before and after the reaction. They show that the particles are uniformly distributed at the GC electrode surface and the particle size is ranging between 70 and 90 nm.

On the other side, the exact composition of the catalyst and the relative surface composition are confirmed by EDS analysis (Fig. 1B and the table inserted therein). In this figure, the characteristic peaks of Pd, Ni, and Sn appear in their position which emphasises that all the components of the nanocomposite are effectively deposited at the GC electrode surface.

Additionally, the crystallographic structure of the prepared nanocomposite is identified using XRD (Fig. 1C). The figure illustrates the XRD patterns of Pd/GC (Fig. 1C, curve a) and PdNiSn/GC (Fig. 1C, curve b). In both curves, a broad peak is observed that extends from 2θ of 20° to 28° which corresponds to C(002) of GC electrode^36^. Moreover, four peaks of Pd metal are obtained at 2θ of ca. 39.7°, 43.2°, 67.2° and 78° related to Pd(111), Pd(200), Pd(220) and Pd(311) planes of face centred cubic (FCC) structure, respectively^33,34^. At PdNiSn/GC electrode (Fig. 1C, curve b), the same peaks appeared at Fig. 1C, curve a are observed in addition to other peaks at 2θ of 19.2°, 33.4°, and 59.4° assigned for Ni(001), Ni(100), and Ni(110) planes of $\beta$-Ni(OH)_2_ of the Ni-containing catalyst^1,37,38^, in addition, one more peak at 2θ of 51.7° related to Sn(211) plane of SnO_2_ is observed. The other three planes of SnO_2_; Sn(110), Sn(101), and Sn(301) appear at 2θ of 26.5°, 33.8°, and 65.8° and overlap with the C(002) peak of carbon, Ni(100) plane of $\beta$-Ni(OH)_2_, and Pd(220) plane of Pd metal, respectively^1^.

### Glycerol Electro-oxidation reaction (GEOR) and capacitance correlation

**Figure 2:**
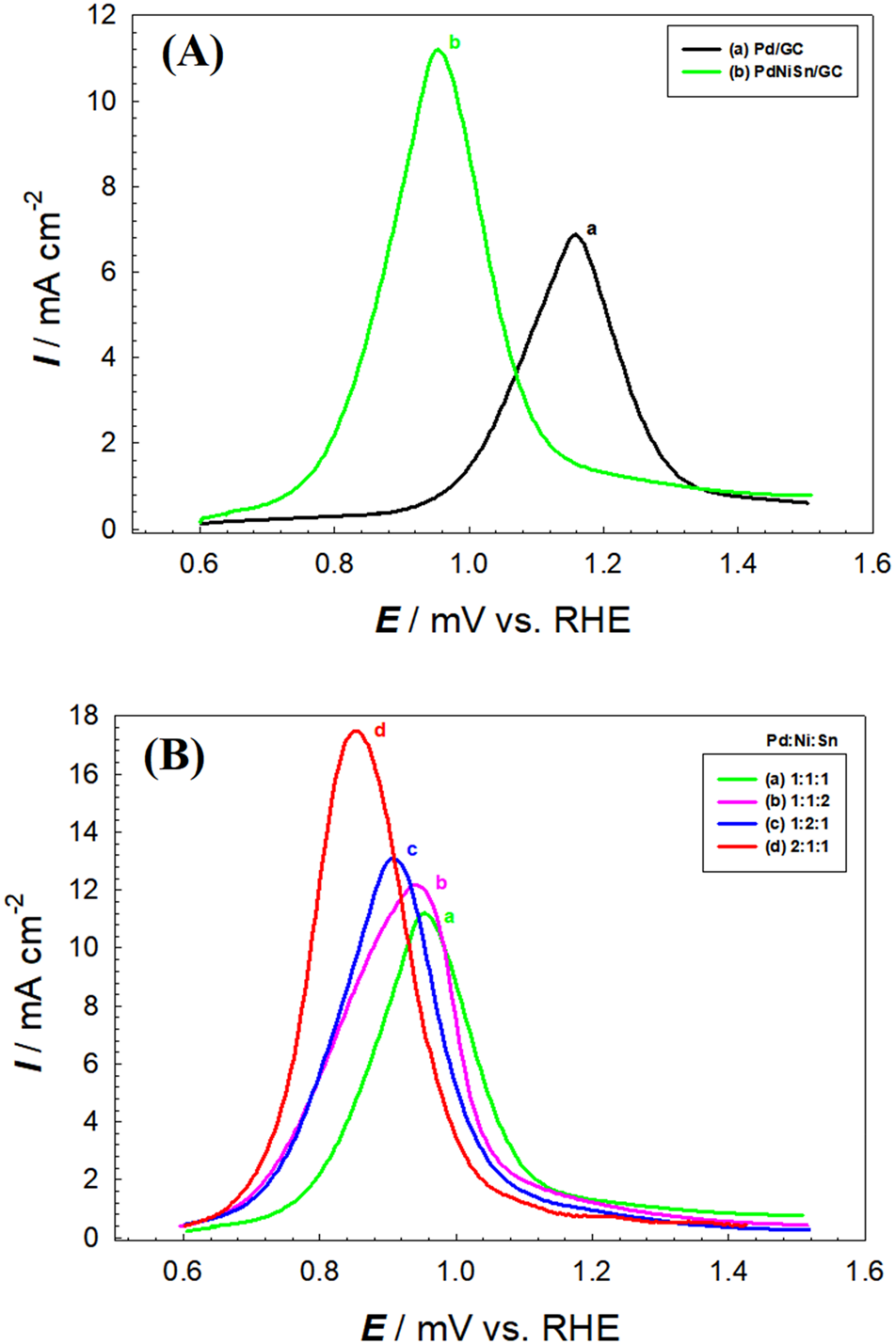
(**A**) LSVs obtained at (a) Pd/GC, and (b) PdNiSn/GC (molar ratio = 1:1:1) (**B**) LSVs obtained at PdNiSn/GC with molar ratio of Pd:Ni:Sn = (a) 1:1:1, (b) 1:1:2, (c) 1:2:1, and (d) 2:1:1 electrodes in 0.1 M KOH containing 0.3 M glycerol. Potential scan rate = 0.05 Vs^−1^.

Figure 2A and Table 1 compare the electrocatalytic activity obtained at Pd/GC (Fig. 2A., curve a) and PdNiSn/GC with molar ratio of Pd:NiSn = 1:1:1 (Fig. 2A. curve b) towards GEOR. The figure and the table illustrate that the peak current (*I*_p_) of GEOR obtained at PdNiSn/GC (Pd:Ni:Sn = 1:1:1) surface is 1.6 times of that obtained at Pd/GC surface plus cathodic shift in the onset potential (*E*_onset_) of GEOR of ca. 200 mV. These results highlight the role of Ni and Sn oxides ($\beta$-Ni(OH)_2_ and SnO_2_) in boosting the ability of Pd to enhance the oxidation of glycerol and the reaction intermediates by increasing the charge transfer in the media.

**Table 1:** Variation of the catalytic enhancement factor and the onset potential of GEOR at the various electrodes in 0.1 M KOH containing 0.3 M glycerol.

Electrode	*E*_onset_^a^/mV	*I*_p_/mA cm^−2^	Enhancement factor^b^
Pd/GC	900	7.0	–
PdNiSn/GC (Pd:Ni:Sn = 1:1:1)	700	11.2	1.6
PdNiSn/GC (Pd:Ni:Sn = 1:1:2)	680	12.2	1.7
PdNiSn/GC (Pd:Ni:Sn = 1:2:1)	650	13.1	1.9
PdNiSn/GC (Pd:Ni:Sn = 2:1:1)	600	17.5	2.5

^**a**^Refers to the onset potential of GEOR estimated at a constant current density of 500 µA cm^−2^ for all electrodes.

^**b**^The enhancement factor for GEOR is calculated by dividing *I*_p_ of GEOR obtained at PdNiSn/GC electrode by *I*_p_ of GEOR at Pd/GC electrode.

Moreover, the optimization of the molar ratio of Pd:Ni:Sn in PdNiSn nanocomposite was crucial. Figure 2B shows the LSVs obtained at PdNiSn/GC with molar ratio of Pd:Ni:Sn = 1:1:1 (curve a), 1:1:2 (curve b), 1:2:1 (curve c), and 2:1:1 (curve d) electrodes in 0.1 M KOH containing 0.3 M glycerol. Figure 2B and Table 1 disclose that the highest electrocatalytic activity of GEOR is obtained when the molar ratio of Pd:Ni:Sn = 2:1:1 (Fig. 2B, curve d), where the *I*_p_ of GEOR is 2.5 of that obtained at Pd/GC plus cathodic shift in *E*_onset_ of GEOR = 300 mV.

Actually, the *I*_p_ value (17.5 mA cm^−2^) obtained at PdNiSn/GC with molar ratio of Pd:Ni:Sn = 2:1:1 towards GEOR is higher than that obtained at similar electro-catalysts in the literature (Table 2). It is about 4 and 3 times of that obtained at polycrystalline Pt incorporated with Pb and Bi (Pt_p_-Pb, Pt_p_-Bi), respectively^39^. Moreover, *I*_p_ obtained at PdNiSn/GC is 15 times of that obtained at 10% Pt/WC and 19 times of that obtained at 10% Pt/TaC^40^. In addition, *I*_p_ obtained at PdNiSn/GC is 7 times of that obtained at PdFe/rGO^41^. On the other hand, it is 35 and 12.5 times of that obtained at Pd/C and PdCu/C, respectively^42^. Furthermore, the electrocatalytic activity obtained at PdNiSn/GC is 13 and 6.5 times of that obtained at Pt and Pd electrodeposited on carbon paper (Pt/CP and Pd/CP), respectively ^43^.

**Table 2:** Variation of the oxidation peak current (*I*_p)_ and onset potential (*E*_onset_) of GEOR at different electrocatalysts in alkaline medium.

Catalyst	Glycerol concentration (M)	Activity		References
		*I*_p_/mA cm^−2^	*E*_onset_^a^/mV	
Pt_p_-Pb (10^–5^ M Pb^2+^)	0.1	4.5	450 vs. RHE	^37^
Pt_p_-Bi (10^–5^ M Bi_2_O_3_)	0.1	6.3	550 vs. RHE	^37^
10% Pt/WC	1.0	1.2	600 mV vs. RHE	^38^
10% Pt/TaC	1.0	0.9	600 mV vs. RHE	^38^
PdFe/rGO	0.1	2.5	700 mV vs. RHE	^39^
Pd/C	3.0	0.5	− 100 mV vs. NHE	^40^
PdCu/C	3.0	1.4	− 100 mV vs. NHE	^40^
Pt/CP	0.1	1.3	− 250 mV vs. NHE	^41^
Pd/CP	0.1	2.6	− 180 mV vs. NHE	^41^
PdNiSn/GC (Pd:Ni:Sn = 2:1:1)	0.3	17.5	600 vs. RHE	This work

^**a**^Refers to the onset potential of GEOR estimated at a constant current density of 500 µA cm^−2^ for all electrodes.

**Figure 3:**
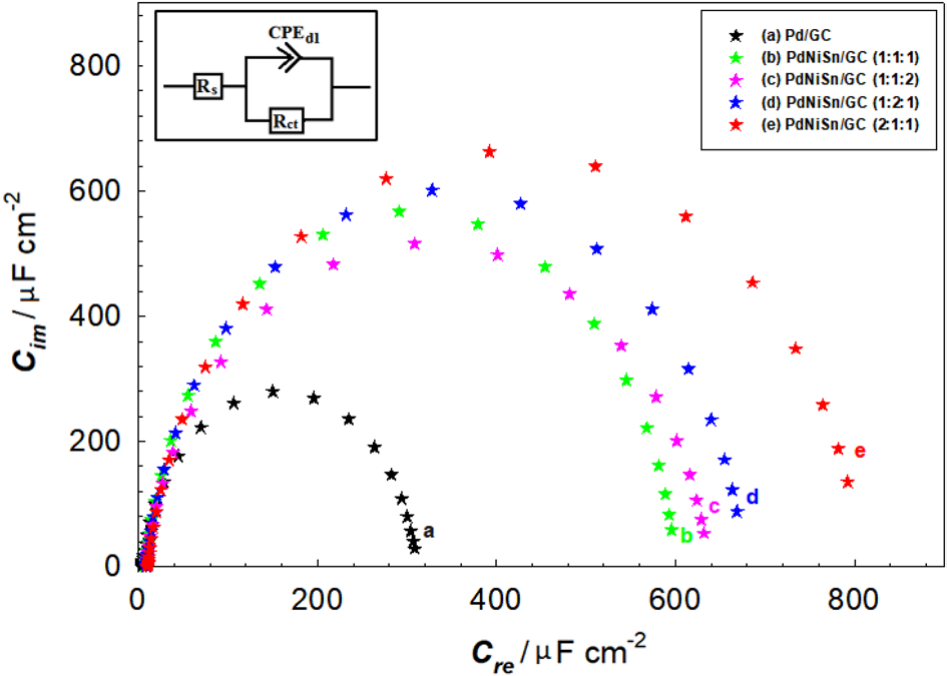
Capacitance curves obtained at (a) Pd/GC, and (b) PdNiSn/GC (molar ratio = 1:1:1), (c) PdNiSn/GC (molar ratio = 1:1:2), (d) PdNiSn/GC (molar ratio = 1:2:1), and (e) PdNiSn/GC (molar ratio = (2:1:1) in 0.1 M KOH containing 0.3 M glycerol . The inset figure represents the equivalent circuit compatible with the results.

The results of the electrocatalytic activity obtained at the ternary structures of PdNiSn towards GEOR were interpreted by correlating this activity with the results of the capacitance obtained at the same electrodes in the same solution. Figure 3 (curves a-e) shows the capacitance curves of Pd/GC (curve a), PdNiSn/GC (molar ratio = 1:1:1, curve b), PdNiSn/GC (molar ratio = 1:1:2, curve c), PdNiSn/GC (molar ratio = 1:2:1, curve d), and PdNiSn/GC (molar ratio = 2:1:1, curve e) in 0.1 M KOH containing 0.3 M glycerol. The figure reveals that the real capacitance (*C*_re_) values obtained at these electrodes are compatible with the values of the electrocatalytic activity of GEOR and the increase in *I*_p_ values (Fig. 2A and B) is consistent with the increase in *C*_re_ values (Fig. 3). For instance, both, *I*_p_ and *C*_re_ values obtained at PdNiSn (2:1:1) is about 2.5 times of those obtained at Pd/GC electrode.

These results clarify that the reason behind the obtained catalysis is due to the catalyst composition and the solution content. In other words, PdNiSn (with molar ratio = 2:1:1) offers a favourable conditions for the adsorption of glycerol molecules at its surface, then, the number of the charged adsorbed species is increased consequently (Fig. 3, curve e), leading to a significant enhancement in GEOR at its surface (Fig. 2B, curve d).

### Stability of PdNiSn/GC electrode towards GEOR

**Figure 4:**
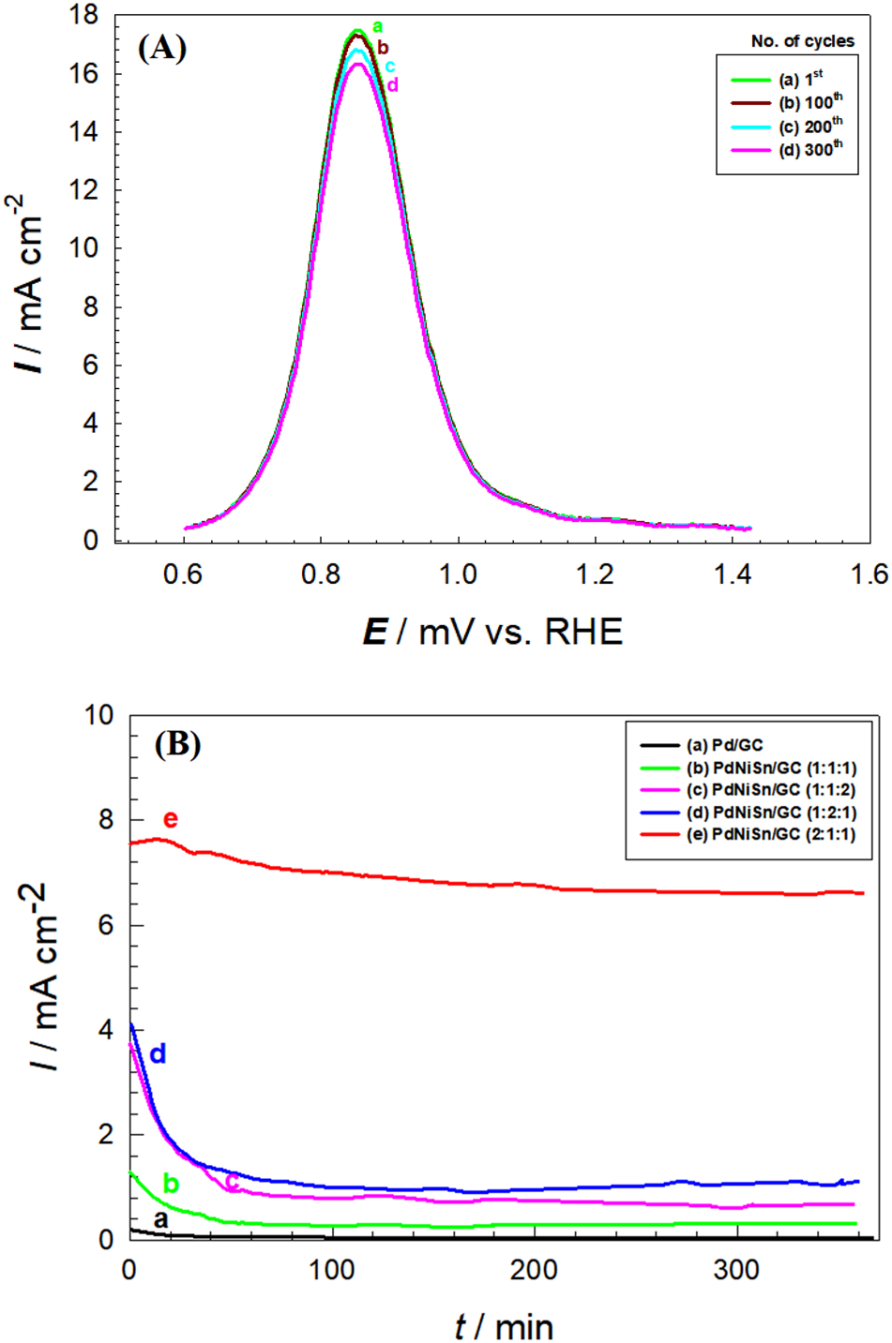
(**A**) LSVs obtained at PdNiSn/GC (molar ratio = 2:1:1) in 0.1 M KOH containing 0.3 M glycerol (potential scan rate = 0.05 Vs^−1^) after ageing for several potential cycles (1^st^, 100^th^, 200^th^, and 300^th^ potential cycles) and (**B**) Current transients (*I-t*, at 0.77 V vs. RHE) obtained at (a) Pd/GC, and (b) PdNiSn/GC (molar ratio = 1:1:1), (c) PdNiSn/GC (molar ratio = 1:1:2), (d) PdNiSn/GC (molar ratio = 1:2:1), and (e) PdNiSn/GC (molar ratio = (2:1:1) in 0.1 M KOH containing 0.3 M glycerol.

Furthermore, PdNiSn/GC (molar ratio = 2:1:1) shows an excellent repeatability and reproducibility. Figure 4A shows that the *I*_p_ of GEOR obtained at its surface remained at almost the same value after 300 potential cycle. In addition, the current transient curves (Fig. 4B) were obtained at Pd/GC (curve a), PdNiSn/GC (molar ratio = 1:1:1, curve b), PdNiSn/GC (molar ratio = 1:1:2, curve c), PdNiSn/GC (molar ratio = 1:2:1, curve d), and PdNiSn/GC (molar ratio = 2:1:1, curve e) electrodes in 0.1 M KOH containing 0.3 M glycerol. They disclose that the overall stability in the current followed similar behaviors among the first four systems studied (Fig. 4B, curves a-d), where the currents obtained at their surfaces diminished to more than half of their initial values at the first 30 min. of the experiment and continued to decay until the end of the hour-long experiment. However, the current obtained at PdNiSn/GC (molar ratio = 2:1:1, curve e) electrode shows a very high stability with just a slight decay in the current obtained at its surface. This reflects the high tolerance of PdNiSn/GC (molar ratio = 2:1:1, curve e) electrode against the poisoning intermediate species by preventing their adsorption or facilitating their oxidation to a final product. The catalyst composition and ingredients’ ratio are believed to be the reason behind the obtained reinforcement in the stability of the current at the electrode surface (Fig. 4B, curve e).

## Discussion

**Figure 5:**
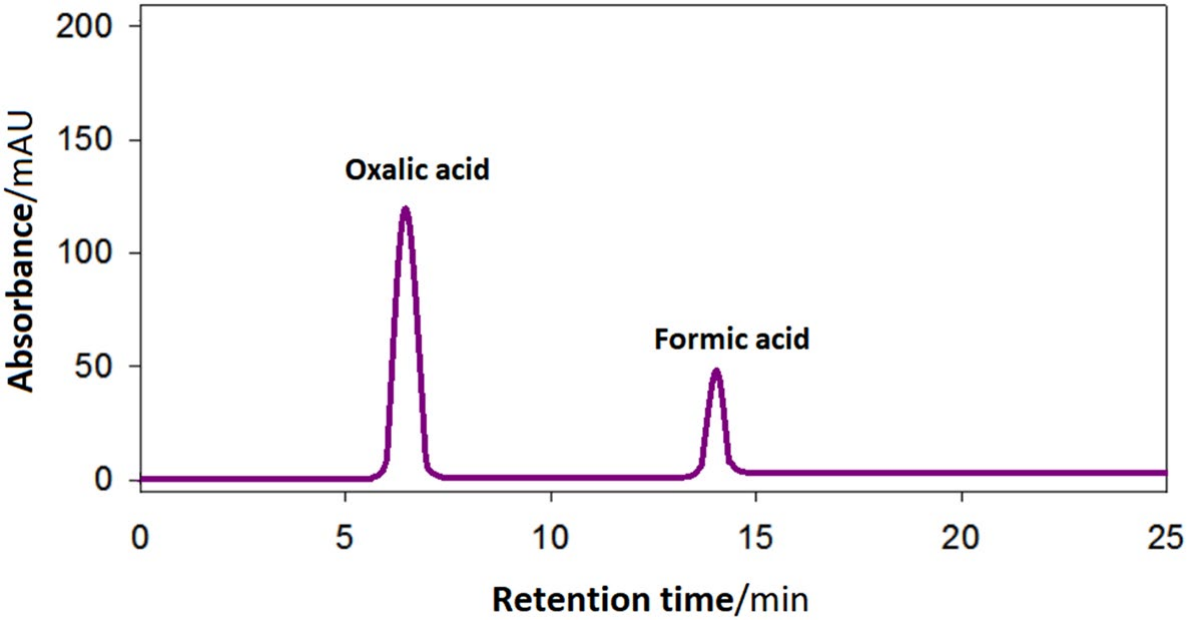
Chromatogram of GEOR product obtained at PdNiSn/GC electrode (molar ratio of Pd:Ni:Sn = 2:1:1) at *E* = 0.77 V vs. RHE for 6 h.

**Figure 6:**
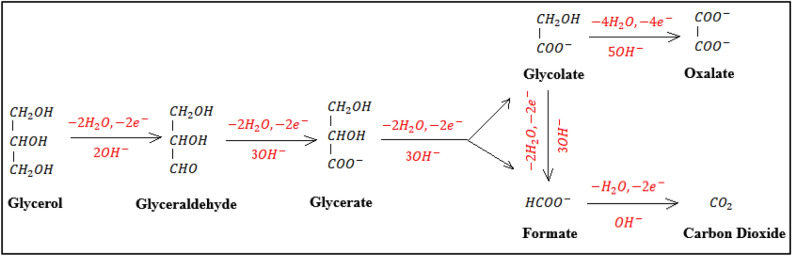
Schematic illustration of the possible oxidation pathways of glycerol in alkaline medium with the corresponding number of exchanged electrons.

Figure 5 identifies the oxidation products obtained from GEOR at PdNiSn/GC electrode after 6 h of accumulation of the products at *E* = 0.77 vs. RHE. It depicts that only two products are obtained; oxalic acid (C2 species, 68%) and formic acid (C1 species, 32%) which highlights the high selectivity of the prepared catalyst to produce two products of interest from GEOR. This result discloses the following: (i) PdNiSn catalyst could efficiently oxidize all the glycerol molecules in the sample, (ii) there are two possible reaction pathways for GEOR at PdNiSn catalyst (Fig. 6), starting from glyceric acid (glcerate); the first one proceeds via the consecutive electro-oxidation of the functional groups without breaking or attacking the C–C bond producing glycolic acid (glycolate) through two electron transfer reaction, then, glycolic acid is further oxidized to oxalic acid (oxalate) through four electron transfer reaction ^41,44^ and the second one proceeds via the cleavage of the C–C bond and the electro-oxidation of the C1 fragments producing formic acid (formate) through two electron transfer reaction^41,44^.

Moreover, the amount of charge (*Q*) consumed during GEOR to oxalic acid (*Q*_oxalic acid_) and to formic acid (*Q*_formic acid_) was calculated using their relative peak areas in the HPLC pattern. These values were estimated as 1.38 and 0.6 C for *Q*_oxalic acid_ and *Q*_formic acid_, respectively. The addition of the two values (1.98 C) is below that recorded for GEOR (*Q*_glycerol_) during glycerol electrolysis (3.6 C). This difference highlights that there is other oxidation product was formed, e.g., formic acid could further been oxidized to CO_2_ which consumes a part of (*Q*_glycerol_). *N.B.*, the percentage (%) of the two acids (oxalic acid and formic acid) in the above calculations were probed using their relative peak intensities in the HPLC pattern for the products of GEOR.

The HPLC results (Fig. 5) together with the charge calculations show that PdNiSn catalyst has high selectivity to CO_2_ through enhancing the electro-oxidation of formic acid and oxalic acid to CO_2_.

These results point towards the synergistic effect between Pd, Ni(OH)_2_, and SnO_2_ in PdNiSn nanocomposite. This synergy enhanced the ability of Pd to cleave the C–C bond and completely oxidize the reaction intermediates.

## Conclusion

The surface of PdNiSn nanocomposite electrodeposited at GC electrode (PdNiSn/GC) offered an efficient electro-oxidation of glycerol in this study. PdNiSn/GC (with a molar ratio of Pd:Ni:Sn = 2:1:1) exhibited both the highest capacitance and the highest electrocatalytic activity towards glycerol electrooxidation reaction (GEOR), when compared to Pd/GC, PdNiSn/GC (molar ratio of Pd:Ni:Sn = 1:1:1), PdNiSn/GC (molar ratio of Pd:Ni:Sn = 1:1:2), and PdNiSn/GC (molar ratio of Pd:Ni:Sn = 1:2:1) electrodes. Furthermore, PdNiSn/GC (molar ratio of Pd:Ni:Sn = 2:1:1) offered the highest current stability among all of the aforementioned electrodes. HPLC analysis revealed that GEOR resulted in the production of two products, oxalic acid (C2 species) and formic acid (C1 species). The results of the HPLC analysis, as well as the charge calculations, indicated that the synergistic effect between Pd, β-Ni(OH)_2_, and SnO_2_ played an essential role in promoting GEOR by enhancing C–C bond cleavage and completely oxidizing the reaction intermediates to CO_2_.

## Data Availability

All data generated or analysed during this study are included in this published article.
